# Metabolic syndrome diagnosis and widespread high grade prostatic intraepithelial neoplasia significantly increase prostate cancer risk: results from a multicenter biopsy study

**DOI:** 10.1186/s12885-016-2085-8

**Published:** 2016-02-04

**Authors:** Antonio Cicione, Cosimo De Nunzio, Andrea Tubaro, Francesco Cantiello, Stefano Manno, Carlos Oliveira, Estevao Lima, Rocco Damiano

**Affiliations:** Magna Graecia University, Viale Europa, 88100 Catanzaro, Italy; La Sapienza University, Via Grottarossa, 00189 Roma, Italy; Braga Hospital, Sete Fontes, São Victor, Braga, Portugal

**Keywords:** High grade prostatic intraepithelial neoplasia, Metabolic syndrome, Prostate cancer

## Abstract

**Background:**

To test in multicenter setting if patients affected of metabolic syndrome (MetS) and initial widespread high grade prostatic intraepithelial neoplasia (wHGPIN) diagnosis are at higher risk of prostate cancer (PCa) on repeat biopsy.

**Methods:**

Patients clinical charts from three European Academic Hospital were reviewed in order to identify patients with initial diagnosis of HGPIN undergone to repeat biopsy. Inclusion and exclusion criteria were adopted to minimize patient heterogeneity. MetS was defined according to Word Heart Organization criteria while initial wHGPIN when ≥4 cores biopsy were involved. A multivariate logistic model was computed to assess the association between PCa and clinical-pathological variables.

**Results:**

Overall 283 patients were scheduled. Median age was 67 years (IQR 62–72). MetS was diagnosed in 116/283 (41 %) patients and PCa was detected in 84/283 (29.7 %) patients.

In particular, PCa was more frequently diagnosed in patients affected of wHGPIN and MetS (45/86, 52.3 %) than in patients with wHGPIN and normal metabolic profile (28/95, 29.5 %), *p* = 0.002. The multivariate logistic model confirmed that wHGPIN and MetS are independent risk factors for following PCa diagnosis, respectively OR 2.4 (95 % CI 1.01–5.71, *p* = 0.04), OR 2.79 (95 % CI 1.49–5.22, *p* = 0.01) while total PSA and DRE findings are not able to predict PCa at repeat biopsy, OR 1.05 (95 % CI 0.98–1.03 *p* = 0.69) and OR 1.01 (95 % CI 0.55–1.84, *p* = 0.96) respectively.

**Conclusions:**

wHGPIN is positively associated to PCa; assessing metabolic profile and repeat prostate biopsy is advisable in patients with initial diagnosis of wHGPIN.

**Electronic supplementary material:**

The online version of this article (doi:10.1186/s12885-016-2085-8) contains supplementary material, which is available to authorized users.

## Background

In 1986 McNeal and Bostwick introduced the pathological diagnosis of high grade prostatic intraepithelial cancer (HGPIN) as a preneoplastic lesion for prostate cancer (PCa). From a histological point of view, it can briefly be described as an atypical epithelial cell proliferation within preexistence acini or ducts with cytological and molecular findings similar to cancer [1].

The clinical meaning of HGPIN is due to an upper risk of subsequent prostate cancer detection. Although this risk decreased to a median of 24 % since the introduction of 12 core template prostate biopsy, it is still higher than the risk to detect PCa following a initial benign diagnosis [2]. Furthermore the current EAU guideline have suggested early repeat biopsy in case of extensive HGPIN diagnosis [3]. This advise is mainly due to the absence of clinical parameters such as prostate specific antigen (PSA), digital rectal examination (DRE) or imaging technique able to identify which patients will have PCa on follow biopsy.

Therefore, new strategies are required to identify patients affected of HGPIN at higher risk of PCa in order to reduce unnecessary prostate biopsies and to elucidate prostate cancer carcinogenesis at same time. Some evidences summarized by De Marzo et al. indicate the chronic prostate inflammation as a potential cause of prostatic cells overgrowth and oncogenesis [4, 5]. In this context, metabolic syndrome (MetS) recognized as an systemic inflammatory condition may act.

Our group has recently reported that patients with HGPIN and MetS are at higher risk of prostate cancer on subsequent biopsy [6]. However this initial finding requires validation by more observations collected on different sets of patients [5].

Thus, the current study is designed to corroborate our previous experience namely whether the diagnosis of metabolic syndrome increases the risk of prostate cancer in patients reporting an initial diagnosis of HGPIN.

## Methods

Clinical charts from three tertiary academic centres were reviewed to identify patients undergone to a repeat prostate biopsy for initial diagnosis of HGPIN between December 2004 and March 2013.

Before initiation, This study was approved by the ethics committee of Magna Graecia University, La Sapienza University-Sant’Andrea Hospital and Braga Hospital that provided the necessary institutional data-sharing agreements. Furthermore, patients had signed a written consent to prostate biopsies and clinical data access for research purpose.

In order to minimize heterogeneity between centers patients, inclusion and exclusion criteria were adopted and all the data were included in a single common datasheet expressly created for the purpose.

The main inclusion criteria was a repeat biopsy for initial diagnosis of HGPIN carried out by 6 months of initial biopsy instead of PSA level and DRE findings. Furthermore a 12 core prostate biopsy scheme under local anesthesia had to be adopted for both biopsies.

Patients receiving 5-Alpha reductase inhibitors, repeat saturation biopsy or prostate transition zone sampling, previous prostate surgery and concomitant diagnosis of atypical small acinar proliferation (ASAP) were excluded from the study.

Furthermore, World Health Organization (WHO) clinical criteria (Table 1) [7] were adopted for diagnosis of metabolic syndrome while widespread HGPIN was defined when it was diagnosed in four or more than four biopsy cores on initial prostate biopsy [8].

**Table 1:** Study criteria for Metabolic Syndrome (MetS) diagnosis

Diagnosis of MetS requires:
Insulin resistance, identified by 1 of the following:
Type 2 diabetes, Impaired fasting glucose, Impaired glucose tolerance,
or for those with normal fasting glucose levels (<110 mg/dL), glucose uptake below the lowest quartile for background population under investigation under hyperinsulinemic, euglycemic conditions
Plus any 2 of the following:
Antihypertensive medication and/or high blood pressure (≥140 mm Hg systolic or ≥90 mm Hg diastolic)
Plasma triglycerides ≥150 mg/dL (≥1.7 mmol/L)
HDL cholesterol <35 mg/dL (<0.9 mmol/L) in men or <39 mg/dL (1.0 mmol/L) in women
BMI >30 kg/m^2^ and/or waist:hip ratio >0.9 in men, >0.85 in women
Urinary albumin excretion rate ≥20 μg/min or albumin: creatinine ratio ≥30 mg/g

Adapted from Grundy SM et al. [7]

Clinical (age, prostate volume, BMI, DRE findings, MetS and widespread diagnosis) and serum variables (total and ratio PSA, HDL cholesterol level, plasma triglycerides and fasting glucose) were analyzed in order to identify significative difference between patients with prostate cancer and benign diagnosis on repeat biopsy.

### Statistical analysis

Data showed a not normal distribution at Shapiro-Wilk test. Whereby, data are presented in median value and interquartile range (IQR: 25^th^–75^th^ percentile) while Mann Whitney and Chi-square test were respectively utilized to test significative level of continuous and categorical variables.

Then to analyze the association between prostate cancer diagnosis at repeat biopsy and analyzed variables a multivariate logistic regression model was used. All reported p value are two-sided an significance was set at 0.05. Analysis was done by SPSS® 18.0 for Windows (SPSS Inc., IBM Corp., Somers, NY, USA).

## Results

**Fig. 1:**
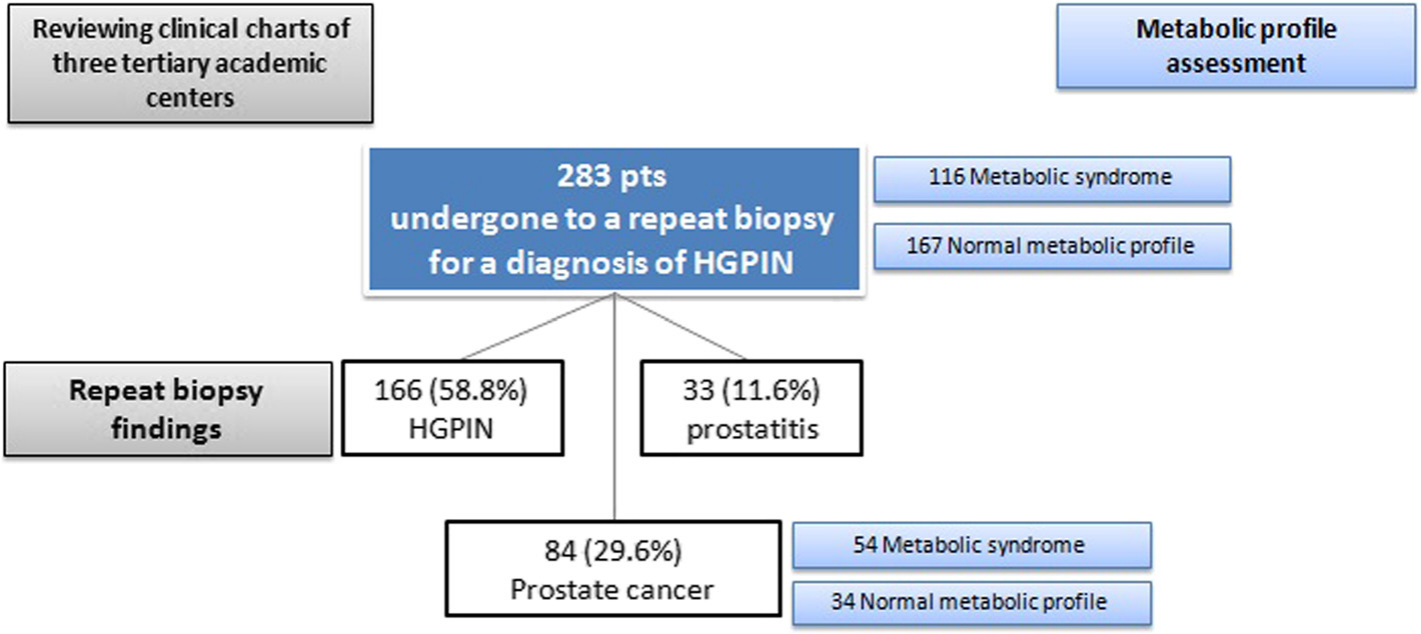
Patients study flow chart

Clinical records of two hundred and eighty three patients affected of HGPIN and undergone to a repeat prostate biopsy were evaluated (Fig. 1).

Diagnosis of prostate cancer was reported in 84 patients (29.6 % of whole cohort) with a Gleason Score respectively Six in 54 (64.6 %), Seven in 20 (24.7 %) and Eight in 10 (12.7 %) patients. In the remaining patients, a diagnosis of HGPIN in 166 patients (58.8 %) and prostatitis in 33 (11.6 %) was reported.

Table 2 shows study population features related to prostate cancer diagnosis or not on repeat biopsy. No significative differences were found between patients with or without prostate cancer in term of age, prostate volume, BMI and DRE findings. Likewise, no significative differences were detected about total and ratio PSA, HDL cholesterol, plasma triglycerides, fasting glucose and hypertensive medication assumption between patients with cancer and benign diagnosis.

However, prostate cancer was more frequent in patients with a previous diagnosis of wHGPIN than isolated HGPIN: 73/181 (40.3 %) Vs 11/102 (10.2 %), *p* = 0.001. Similarly, patients affected of MetS had PCa more recurrent than patients with a normal metabolic status, respectively 54/116 (46.6 %) and 30/167 (18 %), *p* = 0.005. A similar finding was achieved by analyzing also patients with initial isolated HGPIN (≤=3 positive core) (data reported in Table 1S of Additional file 1). Merely analyzing patients with wHGPIN (Table 3), prostate cancer was more frequently diagnosed in patients with MetS than patients with normal metabolic profile, respectively 45/86 (52.3 %) Vs 28/95 (29.5 %), *p* = 0.002. In particular, the diagnosed Gleason Score was Six in 31/45 (68.8 %) and 19/28 (67.8 %), Seven in 10/45 (22.2 %) and 6/28 (21.4 %), Eight in 4/45 (9 %) and 3/28 (10.8 %) patients affected of wHGPIN and MetS or normal metabolic profile, respectively.

**Table 3:** Repeat biopsy diagnosis in patient with initial widespread high grade prostatic intraepithelial neoplasia diagnosis according to their metabolic profile

[No -%]	Prostate cancer diagnosis	No prostate cancer diagnosis
Metabolic syndrome	45/86 (52.3 %)	41/86 (47.7 %)
Normal metabolic profile	28/95 (29.5 %)	67/95 (70.5 %)

Chi-square test: *p* = 0.002

Finally, the regression model confirmed that MetS diagnosis and wHGPIN are predictive variables for subsequent PCa diagnosis (respectively OR 2.79, 95 % IC 1.49–5.22, *p* = 0.01 and OR 2.4, 95 % IC 1.01–5.71, *p* = 0.04) while PSA, PSA free, DRE findings, fasting glucose, blood hypertension status, BMI, plasma triglycerides are not able for that purpose (Table 4).

**Table 4:** Multivariate Regression Model analyzing predictive variables of prostate cancer on repeat biopsy

	Odds ratio	95 % CI		p.
Total PSA	1.05	0.98	1.03	0.69
PSA Ratio	0.99	0.95	1.03	0.70
DRE	1.01	0.55	1.84	0.96
Fasting glucose	1.02	0.98	1.01	0.67
Antihypertensive medication or high blood pressure	1.03	0.92	1.03	0.65
BMI	0.96	0.86	1.05	0.98
HDL cholesterol	0.84	0.72	1.06	0.80
Plasma Triglycerides	0.99	0.93	1.05	0.63
MetS Diagnosis	2.79	1.49	5.22	0.01
Widespread HGPIN	2.40	1.01	5.71	0.04

## Discussion

HGPIN is still considered as a preneoplastic lesion. Epidemiological, molecular and morphological evidences summarized by Montironi et al. underline reasons of HGPIN may be prostate cancer precursor [9]. Albeit the risk of subsequent prostate cancer in patient with initial diagnosis of HGPIN has been reduced to 24 % since the introduction of 10 or 12-core prostate biopsy, it is higher than 18.7 % after a benign diagnosis [2]. For this reason, EAU guidelines suggest to perform a new early biopsy in case of extensive HGPIN diagnosis [3] however it doesn’t suggest when.

This study confirms our previous result in a larger sample: patients affected of widespread HGPIN and Metabolic Syndrome are at higher risk of prostate cancer [6]. A chronic inflammatory environment due to MetS may explain this result. De Marzo et al. [4] proposed that chronic prostatic inflammation may led to prostate cancer thought progressive lesions where HGPIN may be the middle step. Obesity, diabetes or insulin resistance and dyslipidemia are conditions linked to inflammatory status by pro-inflammatory cytokines secretion [10, 11]. For instance, leptin and interleukin-6 showed in vitro to have a supporting activity in prostate cancer cell lines inducing proliferation, inhibiting apoptosis [12, 13] and increasing migration [14]. Those bioactive substances are secreted by adipose tissue with a well recognized pro-inflammatory activity. Furthermore, they have also been found in high concentration in patient affected of HGPIN without prostate enlargement (prostate gland less than 40 ml) [15] supporting the hypothesis of chronic prostatic inflammatory as possible reason for carcinogenesis. In particular, the consequential inflammatory microenvironment, produces reactive oxygen and nitrogen radicals. These radicals may produce permanent genomic alterations in cellular DNA including neoplastic changes [5]. Indeed, HGPIN shares a number of somatic genetic and epigenetic alterations with PCa. For instance, about 20 % of HGPIN lesion harbour TMPRSS2-ERG fusion gene, which is a common molecular abnormality detectable in about 50 % of prostate cancers [16] as well as aneuploidy which is more frequent in HGPIN than benign prostate tissue, although somewhat less than seen in some invasive carcinomas [9].

In contrast to our previous study, each WHO metabolic syndrome criteria has been also analyzed. It is interesting to be noted that neither BMI, fasting glucose, HDL and triglycerides level are independent PCa risk factors on multivariate analysis whereas having MetS diagnosis increases about two times that risk than patient with normal metabolic profile. To date, MetS is a cluster of disorders and the true mechanism developing cancer has to be known yet. Likely, it may be more complex and be wary of the easy total sum from each metabolic abnormalities consequences [10].

The main study limitation is its retrospective nature. Of course, long prospective comparative study is thought as the best method to evaluate a disease risk factor. Furthermore, including a hormonal profile assessment in such prospective study would be ideal in order to have a deeper understanding of likely cancer development way. However, this was an analysis of prospectively collected data and the same biopsy sampling and method were used in all the centers. Then, initial finding requires validation by more observations, collected on different sets of patients from different centers and retrospective study may be a quick way for this [5]. In this context, the large sample has enabled to corroborate previous results. First, Metabolic syndrome may be considered a high risk factor for subsequent diagnosis of prostate cancer in patients with an initial diagnosis of HGPIN. Then, widespread HGPIN is linked to higher risk of prostate cancer at repeat biopsy. Lee et al. [17] reported an increased risk of prostate cancer in patients affected of diffuse and bilateral HGPIN. Merrimen JL [18] and De Nunzio [19] showed as the risk of subsequent prostate cancer is higher in case of a widespread HGPIN. However the small sample was the main limit in each of those studies.

Finally, the lack of single uro-pathologist reviewing all specimens is a further limit curbing our study findings as well as absence of data on the number of biopsy core positive in PCa.

As well as previous studies [2, 6, 17–19], neither total and ratio PSA level and DRE findings are able to define patients at higher PCa risk on subsequent biopsy.

Currently, there is not a single serum marker able to find out patients with HGPIN at higher risk of subsequent PCa therefore new basic and clinical studies are advisable to reduce the number on unnecessary prostate biopsies. Our data suggest to asses metabolic profile in patient with HGPIN diagnosis and repeat prostate biopsy in case of metabolic syndrome is detected. Furthermore, our data prompt to assess if MetS and wHGPIN may be thought as negative prognostic biopsy factors of PCa since this cancer may be more aggressive in patients affected of MetS [10].

However, further basic and very long prospective studies with careful interest on medications used for metabolic syndrome are needed to clearly understand the true association MetS- HGPIN- prostate cancer and to investigate the possible protective or therapeutic role of such medications.

## Conclusions

Patients with an initial diagnosis of widespread HGPIN and metabolic syndrome look to be at elevated risk of subsequent prostate cancer diagnosis.

This study suggest that metabolic syndrome acts as likely prostate cancer promoting factor at time of preneoplastic lesion too. Furthermore, widespread HGPIN as higher risk factor for subsequent prostate cancer diagnosis has also been confirmed in this large cohort study.

Then, repeating biopsy within six months from the previous one may be a advisable way to manage patients affected of wHGPIN as well as assessing metabolic profile may be a further valid tool to identify patients at higher PCa risk in order to reduce the number of unnecessary prostate biopsies.

### Availability of supporting data

Patients dataset may be request by email to corresponding author without any direct or indirect patients identifiers.

## Additional file

Additional file 1: Table S1. Table reporting repeat biopsy diagnosis in patients with initial isolated (≤3 positive core) HGPIN diagnosis according to their metabolic profile. (DOCX 11 kb)

## Abbreviations

HGPIN: high grade prostatic intraepithelial neoplasia
MetS: metabolic syndrome
PCa: prostate cancer
wHGPIN: widespread high grade prostatic intraepithelial neoplasia
