# Supplementary material for: Metabolic syndrome diagnosis and widespread high grade prostatic intraepithelial neoplasia significantly increase prostate cancer risk: results from a multicenter biopsy study
Source: BMC Cancer. 2016 Feb 4;16:59. doi: 10.1186/s12885-016-2085-8 (PMC4743098; doi:10.1186/s12885-016-2085-8)
Supplement: Additional file 1: Table S1. — Table reporting repeat biopsy diagnosis in patients with initial isolated (≤3 positive core) HGPIN diagnosis according to their metabolic profile. (DOCX 11 kb) [file 12885_2016_2085_MOESM1_ESM.docx]

Additional file 1: Tables S1. Repeat biopsy diagnosis in patient with initial isolated (≤3 positive core) high grade prostatic intraepithelial neoplasia diagnosis according to their metabolic profile

| *[No -%]* | Prostate cancer diagnosis | No prostate cancer diagnosis |
| --- | --- | --- |
| Metabolic Syndrome | 9/30 (30%) | 21/30 (70%) |
| Normal metabolic profile | 2/72 (2.8%) | 70/72 (97.2%) |

Chi-square test: p=0.0001
